# Information use by humans during dynamic route choice in virtual crowd evacuations

**DOI:** 10.1098/rsos.140410

**Published:** 2015-01-21

**Authors:** Nikolai W. F. Bode, Armel U. Kemloh Wagoum, Edward A. Codling

**Affiliations:** 1 Department of Mathematical Sciences, University of Essex, Colchester CO4 3SQ, UK; 2 Department of Engineering Mathematics, University of Bristol, Bristol BS8 1UB, UK; 3 Jülich Supercomputing Centre, Forschungszentrum Jülich GmbH, 52428 Jülich, Germany

**Keywords:** decision-making, crowd evacuations, virtual environment, route choice, Bayesian model selection

## Abstract

We conducted a computer-based experiment with over 450 human participants and used a Bayesian model selection approach to explore dynamic exit route choice mechanisms of individuals in simulated crowd evacuations. In contrast to previous work, we explicitly explore the use of time-dependent and time-independent information in decision-making. Our findings suggest that participants tended to base their exit choices on time-dependent information, such as differences in queue lengths and queue speeds at exits rather than on time-independent information, such as differences in exit widths or exit route length. We found weak support for similar decision-making mechanisms under a stress-inducing experimental treatment. However, under this treatment participants were less able or willing to adjust their original exit choice in the course of the evacuation. Our experiment is not a direct test of behaviour in real evacuations, but it does highlight the role different types of information and stress play in real human decision-making in a virtual environment. Our findings may be useful in identifying topics for future study on real human crowd movements or for developing more realistic agent-based simulations.

## 2. Introduction

When moving within a pedestrian crowd, individuals have to continuously make movement decisions at different temporal and spatial scales. At a microscopic level, individuals have to respond to their immediate environment, to avoid collisions with other pedestrians or obstacles, for example. This has been termed ‘operational level’ of pedestrian behaviour [1]. Empirical and theoretical research suggests that at this level, individuals seek to optimize their travel time or the directness of their route towards a target, while avoiding collisions [2,3]. Operational-level behaviour alone can explain many phenomena of human crowd dynamics, such as the spontaneous formation of lanes in bidirectional pedestrian flows or pedestrian jams at bottlenecks [4]. However, individuals typically also have to make higher level movement decisions, such as choosing between different routes to exit a building. Understanding this ‘tactical level’ of pedestrian behaviour [1] is important, as it determines the distribution of pedestrian crowds at larger spatial scales. For example, the weather protection offered by routes or the length of routes have been suggested to determine pedestrians' route usage in built environments, such as railway stations or city centres [5].

The research presented here is motivated by the tactical-level route choices individuals may have to make in crowd evacuations. Evacuations of human crowds from confined spaces, such as buildings or vehicles, are a paradigmatic example for why understanding tactical-level pedestrian behaviour is important: individual decisions on when to evacuate and which emergency exit route to use determine the distribution of pedestrians across evacuation routes and operational-level behaviour can subsequently lead to potentially dangerous crowd dynamics [4,6]. To give a simple example, the majority of evacuees could choose the shortest exit route from a building, leading to a high pedestrian density along this route (tactical level). High pedestrian densities could subsequently lead to a build-up of pressure at bottlenecks in the evacuation route or density waves increasing the risk for pedestrians to fall (operational level).

It is generally accepted that the length of routes and the degree to which they are congested by other pedestrians are likely to be important aspects in route choice [7–9]. There also seems to be consensus that pedestrians tend to choose the quickest route [7–9]. However, it is not clear how pedestrians determine the quickest route from their own individual perspective and how this process should be represented mechanistically in models for human behaviour [7–9]. One of the key questions in this regard is to what extent individuals rely on static information from the environment, such as exit widths or route length, or on dynamic information, such as the level of congestion along different routes [9,10]. Previous work has established that the likelihood of pedestrians to choose particular routes is influenced by static information, such as the length of the route and the presence of stairs or escalators [5,11]. There is also limited empirical evidence suggesting that pedestrians take the movement of others into account when choosing routes [12,13]. However, to date there is no detailed understanding of the dynamic, time-dependent route choice mechanisms in humans. There is therefore a need to investigate what information individuals rely on to make tactical-level route decisions: static aspects of the environment or dynamic aspects of the environment or both.

In this study, we use an established methodology to conduct experiments on human route choices in virtual environments [14,15]. Such computer-based experiments are an important and well-established tool in research and are used to study diverse topics in behavioural ecology [16], human visual perception [17], as well as in human decision-making, in general [18,19], and in evacuations in particular [13,20,21]. However, we stress that it is important not to over-interpret findings from this type of research. To date, it has not yet been established to what extent human decision-making in virtual environments (including the experiment presented here) extends to the real world. Our experiment is not designed to be a direct test of real pedestrian dynamics in a real environment. Instead, we suggest our work contributes in three ways to our understanding of human decision-making and crowd behaviour. Firstly, in our computer-based experiment we are able to fully and securely control the environment participants perceive in a way that is not possible in experiments with real crowds. This allows us to investigate particular aspects of human route choice in isolation and our findings thus present novel insights into real human decision-making, albeit limited to this specific context. Secondly, experiments with large real crowds are typically expensive and time consuming and obtaining detailed data presents logistical and technical difficulties [4,6]. There are many different scenarios in crowd evacuations that merit our attention. Our approach arguably offers a fast and cost-effective way to develop an intuition for what aspects of behaviour could have important effects on evacuation dynamics. Therefore, our findings could aid in the selection of topics that should be studied in more realistic experiments in real environments. In addition, our findings could also potentially be used to inform the formulation of theoretical crowd movement models [4,6]. Thirdly, the data analysis methods we present here can be applied to data obtained from experiments with real crowds or even real emergencies, and this part of our work is therefore generally applicable.

**Figure 1.**
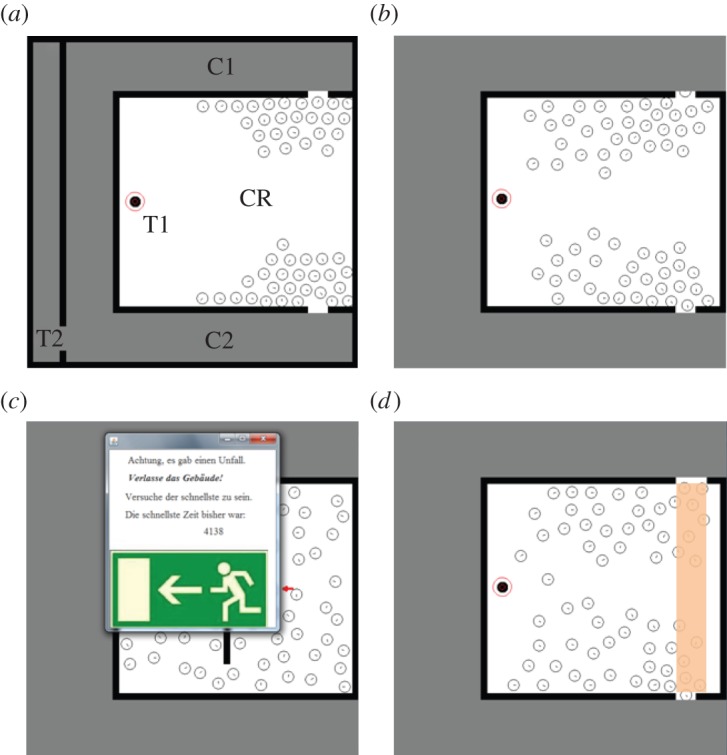
Layout of virtual environment and experimental treatments. (*a*) shortest path treatment, S. The virtual environment comprises a central room (labelled ‘CR’) and two corridors (‘C1’ and ‘C2’) leading to an exit from the virtual environment (‘T2’). Corridor C1 is longer than C2. The experiment consisted of two consecutive tasks. In the first task, participants started at a position to the right of the ‘CR’ label and followed arrows to the first target (‘T1’) to familiarize themselves with the controls in the virtual environment. The second task simulated a crowd evacuation and participants started at ‘T1’, subsequently exited ‘CR’ into either corridor ‘C1’ or ‘C2’ and moved to the final target ‘T2’. Simulated pedestrians are represented by white filled circles with a line indicating their movement direction and the pedestrian steered by participants is represented by a black filled circle, located at ‘T1’. (*b*) control treatment. Compared to (*a*), the global layout of the environment is not visible. (*c*) Motivation treatment, M (message translates to: ‘Attention, there has been an accident. Leave the building! Try to be the fastest. Currently, the fastest time is: 4138’). (*d*) Exit width treatment, W. The top exit, leading into the longer corridor, C1, is 1.5 times as wide as the lower exit, as highlighted by the transparent bar between the two exits. The simulated crowd splits approximately evenly between the two exits in all treatments. All experimental procedures are described in detail in the electronic supplementary material, text.

In our experiment, individual participants had a top-down view of a virtual environment that consisted of a central room and two corridors of unequal length that led to an exit point (figure 1*a*). Participants steered one pedestrian inside this environment with mouse clicks. After familiarizing themselves with the steering of this pedestrian, participants took part in a simulated crowd evacuation and had to choose which of the two possible exit routes to take. Both exit routes were used by computer-simulated pedestrians. In the control condition, participants could not perceive any difference between the two exit routes, even though one route was shorter than the other. Our experimental treatments introduced differences between the two exit routes by either providing participants with information about which route was shorter (‘shortest path’ or S treatment; figure 1*a*) or by increasing the width of the exit leading to the longer route (‘door width’ or W treatment; figure 1*d*). Initially, similar numbers of simulated pedestrians used the two exits, but the latter treatment, W, had the effect that the queue in front of the wider exit disappeared faster. We recorded data on the movement and mouse clicks of participants during the evacuation. All participants in our experiments provided informed consent before starting the experiment.

Emergency evacuations are often highly stressful for evacuees. Previous research suggests that stress affects human decision-making more generally [22,23] and could be important in route decisions during evacuations in particular [14]. Studying the effect of stress experimentally in close proxies for emergency evacuations, such as evacuation drills with volunteers, could potentially be dangerous and result in injuries for participants. This raises ethical questions about such experiments. By contrast, the use of a virtual environment allowed us to safely put participants under additional pressure during experiments (‘motivation’ or M treatment; figure 1*c*). While our approach does not establish exactly how humans behave under stress in real evacuations, it nevertheless allows us to develop an intuition for how time pressure affects real human decision-making.

In summary, we conducted experiments on tactical-level human route choice in crowd evacuations using an interactive virtual environment. In contrast to previous work, we explicitly investigated time-dependent route choice mechanisms in humans and tested whether individuals rely on time-dependent or time-independent information. In addition, we investigated whether additional pressure on participants affected route choice mechanisms.

## 3. Changes in environment affect route choices

We first tested the effect of our primary experimental treatments (motivation, M; exit width, W; shortest path, S) on the proportion of participants who chose the shortest route in the simulated evacuations, *P*(shortest route), and the proportion of participants who changed their mind on which exit to use during the evacuation, *P*(change). We recorded a change of mind for participants if they walked for at least one-fifth of the height of the central room towards one exit before changing direction and exiting through the opposite exit. We assessed the significance of the effect of each treatment on *P*(shortest route) and *P*(change) by conducting single-parameter tests on generalized linear model fits to the data (see the electronic supplementary material, text). For our experiment, we recruited 464 volunteers, who were split up evenly across the control condition (figure 1*b*), the primary experimental treatments (figure 1*a*,*c*,*d*), pairwise combinations of treatments and a combination of all treatments (eight different experimental conditions in total; see the electronic supplementary material, figure S1). Combining treatments allowed us to test how people trade off different types of information. For example, combining treatments S and W leads to a trade-off between using the shorter route and the wider exit. We were particularly interested in combining treatment M with the other treatments, as it provides insights into the effect of additional pressure on participants' responsiveness to features of the environment (e.g. difference in door widths) and to possible changes in crowd movement dynamics (e.g. differences in queue lengths).

In the absence of treatments (control treatment), participants could not see the shortest route and, as expected, we found that in this situation participants selected the shorter route no more frequently than expected by chance: 29 out of 58 participants experiencing the control treatment chose the shorter route (binomial test, *p*=1; electronic supplementary material, figure S1). When the shorter route was visible (treatment S), we expected that participants would be more likely to choose this route. However, treatment S did not have a significant effect on *P*(shortest route) (*p*=0.83; table 1 and electronic supplementary material, table S2). Under treatment W, the exit opposite to the shorter route was wider than the other exit. We found that treatment W significantly reduced *P*(shortest route) (*p*=0.0003; table 1 and electronic supplementary material, table S2). Therefore, participants showed a strong preference for a wider exit, but not for a shorter exit route in our experiment. It is possible that the implementation of treatment S meant that participants did not grasp which exit route was shorter and we will further discuss such aspects of our experiment below. In our experiment, treatment M and gender had no statistically significant effect on *P*(shortest route) (*p*=0.12 and *p*=0.86, respectively; table 1 and electronic supplementary material, table S2). Interestingly, our model fit showed that age had a significant positive effect on *P*(shortest route), suggesting that older participants were more likely to use the shorter route (*p*=0.04; table 1 and electronic supplementary material, table S2). However, this does not necessarily imply that age affected the way participants reacted to our treatments. To further investigate this issue, we extended our statistical model by including interaction terms of age with one treatment, separately for each primary treatment. We found that none of the interactions had a significant effect on *P*(shortest route) (likelihood ratio tests; interaction terms of age with: M—*χ*_(1)_^2^ = 0.07, *p*=0.79; with S—*χ*_(1)_^2^ = 0.00003, *p*=0.99; with W—*χ*_(1)_^2^ = 0.21, *p*=0.65). We suggest that the distribution of ages (strongly centred on the median of 23 years with only about 5% of participants older than 35) does not allow us to establish subtle age-related effects conclusively.

**Table 1.** The effect of the primary treatments, participant age and gender on the proportion of participants choosing the shortest route, *P*(shortest route), and on the proportion of participants who changed their mind on which exit to use during the evacuation, *P*(change). We indicate the effect explanatory variables had on the summary statistics and show the *p*-values of single-parameter tests on binomial generalized linear model fits to the data. Full details can be found in the text and in the electronic supplementary material, tables S2 and S3. Significant values are shown in bold.

symbol	short description	effect on *P*(shortest route)	effect on *P*(change)
S	shortest route is visible	decrease, *p*=0.83	increase, *p*=0.69
W	one exit is wider than the other	**decrease**, ***p***=0.0003	increase, *p*=0.10
M	motivation message displayed	increase, *p*=0.12	**decrease**, ***p***=0.048
gender	gender of participants	decrease, *p*=0.86	increase, *p*=0.21
age	age of participants	**increase**, ***p***=0.04	decrease, *p*=0.31

Previous work suggests that only few participants change their mind on which exit to use during the evacuation (i.e. *P*(change)≪0.5 [14,15]) and indeed we found that across all eight experimental conditions, only 47 out of 464 participants changed their mind. We also expected that treatment M would reduce *P*(change) [14] and found that this was the case (*p*=0.048, table 1 and electronic supplementary material, table S3). Treatments S and W introduced an asymmetry into the virtual environment that made one exit route favourable or led to a trade-off between a shorter route and a wider exit. Participants may not observe this asymmetry immediately or only notice a difference between exits as a result of simulated pedestrians' movement. Therefore, we expected that treatments S and W would increase *P*(change). We found that this was the case for both treatments, but that neither of the effects was statistically significant (electronic supplementary material, table S3). This corroborates our observation from above that treatment S had a weak effect on participants' decisions. We found that age and gender did not have a significant effect on *P*(change) in our data (*p*=0.31 and *p*=0.21, respectively; table 1 and electronic supplementary material, table S3). Table 1 and electronic supplementary material, figure S1, show a summary of our findings.

This analysis confirmed that experimental treatments could affect participants' route choices and therefore provided some insights into individuals' decision-making process: participants tended to prefer wider exits, their decisions were somewhat affected by treatment M, but not by seeing a shorter exit route. Overall, participants were likely to stick with their first choice of exit, and this tendency was stronger under treatment M. However, this preliminary analysis does not allow us to distinguish whether participants react to a difference in door widths or a difference in queue lengths or queue speeds under treatment W. The model selection approach presented in the next section addresses this point.

## 4. Individuals use dynamic instead of static information

We propose novel explanatory models for human route choice to develop a more detailed understanding of the information that participants used in their decisions. By employing state of the art Bayesian model selection, we compared the extent to which the different models were supported by our data [24]. The key difference in the models' structure compared to the statistical models in our preliminary analysis was that instead of predicting the final outcome of individuals' exit choices, the models predicted the probability for individuals to choose either exit over time, explicitly taking into account changes in the environment, such as the movement of simulated pedestrians. The movement and mouse clicks of participants in the virtual environment clearly indicate a decision to move towards one exit. As a starting point, we only considered the situation when participants had already indicated an initial decision for one exit and assumed that participants always displayed a preference for one of the two exits and were therefore never undecided. Our model selection did not take differences in response time into account. For this analysis, we used data from the virtual environment for each participant sampled at fixed intervals resulting in 20.45 data points per participant, on average (for details see the electronic supplementary material, text).

Our analysis was focused on investigating which out of two time-independent and two time-dependent features of the virtual environment best explained participants' exit choices. The two time-independent features were the difference in door widths and the difference in exit route length (when visible, under treatment S) between the two exit routes. The two time-dependent features related to the simulated pedestrian queues in front of the exits and captured the difference in their length (Q) and the difference in the rate of change in their length (‘flow’, F). In other words, the former assumed individuals looked at the length of queues and the latter assumed individuals looked at how fast queues were moving. It is evident that the difference in door widths, W, caused differences in the two time-dependent features Q and F, but it is not clear what information participants were likely to use when making decisions. To test this, we constructed one model for each possible combination of the four factors (W, S, Q, F) and compared the support of the data for these different models, as described below. This led to 16 separate models: one model including all factors, four models including three factors, six models including two factors, four models including only one factor and one model without any of the factors included as a baseline for comparison (see the electronic supplementary material, text and table S1 for implementation details).

We also included three additional factors that could influence participants' exit choices in all models. First, we included a constant, to test for a consistent bias in participants' decisions. Second, for all time points, we considered the effect of how close participants were to one exit compared to the other exit, to test whether participants were less likely to change their decision the closer they were to an exit. Third, we explored whether being ‘jammed’ in a queue affected participants decisions. We might expect that participants were unlikely to change their decision when their path to the opposite exit was blocked by simulated pedestrians. While we were interested in the effect of these additional factors on route choices, we did not make them subject of our model selection and therefore included them in all models.

To determine which models were best supported by our data, ***D***, we computed, for each model, *X*, the marginal likelihood of the data, *P*(***D***|*X*), conditioned on the model for exit route choice (following, e.g. [24]). Given a prior parameter space, the marginal likelihood indicates how likely a model *X* is and it penalizes models with more parameters, as less probability can be assigned to any parameter value *a priori* in the integration over the prior parameter space (for a more detailed discussion see electronic supplementary material, text; [24] and references therein). Each model represents a hypothesis for the decision-making process of participants and we can compare different models *X*_*i*_ and *X*_*j*_ by comparing their marginal likelihood using the Bayes factor, BF=*P*(***D***|*X*_*i*_)/*P*(***D***|*X*_*j*_) [25]. Values of BF>1 imply that model *X*_*i*_ is more strongly supported by the data than model *X*_*j*_. We used a commonly adopted scale of interpretation for BF values: 2log(BF)=0–6, weak to positive evidence; 2log(BF)=6–10, strong evidence; 2log(BF)>10, decisive evidence in support of model *X*_*i*_ [25]. We first considered data in the absence of treatment M and focused our analysis on the effect of two time-independent and two time-dependent aspects of the virtual environment on participants' exit choices.

**Figure 2.**
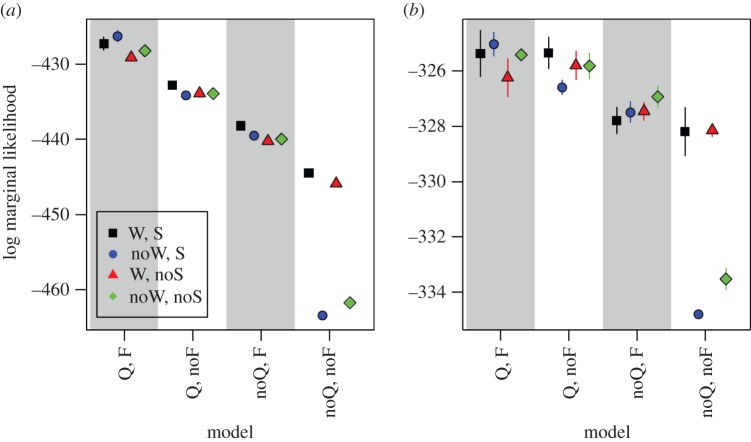
Model selection results. We show the marginal likelihood for each model averaged over five numerical model fitting replicates for the data in the absence (*a*) and presence (*b*) of the M treatment. Error bars show 1 s.d. and are often smaller than the symbol size. Grey bars serve to separate ‘blocks’ of models (see main text). We construct models by accounting for different aspects of the virtual environment that are captured by two time-independent model components (W, exit width; S, exit route length) and two time-dependent model components (Q, queue length; F, queue speed). For example, the first model on the left-hand side in (*a*) (Q, F, W, S), includes all four model components, and the model to its right (Q, F, noW, S) includes all model components, apart from W.

Figure 2*a* and electronic supplementary material, table S4, show the results of our model fitting. From figure 2*a*, we can see that two models had a much lower marginal likelihood than all other models (the next closest model had much stronger support, 2log(BF)=31.6). Both of these models assume that individuals do not take differences in exit width (W), queue length (Q) or queue speed (F) into account when choosing an exit. The weak support of these models when compared with other models was expected from our preliminary analysis above: we found a strong effect of treatment W and therefore we would expect that at least one of the model components relating to W, Q or F needed to be included in models. Figure 2*a* also shows that our analysis did not simply support the most complex model (i.e. the model with the most parameters). For example, the model with all four components W, S, Q and F included had a lower marginal likelihood than the model that included S, Q and F, but not W (figure 2*a*; electronic supplementary material, table S4).

Our approach allowed us to establish which out of the three model components (and associated hypotheses for human behaviour) W, Q and F is most supported by our data. From left to right in figure 2*a*, we first include both Q and F components, second we remove F but keep Q, third we remove Q but keep F and fourth we remove the model components relating to both Q and F. We find a global trend of a decrease in marginal likelihood following this procedure. If we compare the model with the highest marginal likelihood in one ‘block of models’ (within ‘blocks’ we additionally include or exclude components W and S; model blocks are indicated with grey/white stripes in figure 2) to the model with the lowest marginal likelihood in the block immediately to the left, we find strong evidence in support of model blocks to the left (all 2log(BF)>7; e.g. compare model ‘Q, F, W, noS’ (first block) to model ‘Q, noF, W, S’ (second block): 2log(BF)=7.2, from electronic supplementary material, table S4). Therefore, regardless of whether components W and S are included in our models, we find that there is strong support for including both Q and F in our models. In other words, our analysis suggests that participants were more likely to have based their exit choices on differences in queue lengths and queue speeds at the two exits than on differences in exit widths. As expected from our preliminary analysis in the previous section, including the model component S to take differences in path length (if visible) into account did not have a pronounced effect on the marginal likelihood of models.

By looking at values of the most likely parameters for our models, we can understand how the different model components affected participants' exit choices. We found that the parameter which indicates the strength of the effect of the model component S (shorter route) was typically positive (parameter *p*_2_, electronic supplementary material, table S4). This suggests that when the shorter route was visible, participants were more likely to choose this route (compare to implementation of models in the electronic supplementary material, table S1 and equation S1, text). Likewise, we found parameter values suggesting that participants were more likely to choose the wider exit, the exit with the shorter queue and the exit with the faster moving queue (parameters *p*_3_, *p*_4_ and *p*_6_, respectively). Only in models that included both Q and F did we find parameter values suggesting that participants chose narrower exits. However, this last, somewhat unexpected parameter value has to be viewed in the context that components Q and F capture participants' decisions better in these models. The value of the constant effect indicates whether there was a bias for participants to choose one of the exits. In a perfectly symmetric environment, we would expect that participants do not show a preference for either exit, suggesting values of the parameter for the constant effect close to zero. In practice, we find relatively low values and deviations from zero have to be viewed in the context of the model structure and the values of other parameters (parameter *p*_1_, electronic supplementary material, table S4).

Throughout, parameter values showed that the closer participants got to one exit, the more likely they were to stick with their decision for this exit (parameter *p*_8_ in the electronic supplementary material, table S4). It is possible that participants only display this behaviour once they have made an initial investment of a certain size by walking towards one exit. However, we found no relevant threshold for differences in distances to the exits on average (parameter *p*_9_ in the electronic supplementary material, table S4). Across all data used in this analysis, approximately 15% of data points captured a situation when participants were blocked inside a queue in front of one exit. We found that at most four simulated pedestrians at a time blocked the path of a participant. Our most likely parameter estimates show that being blocked in a queue essentially determined participants' decision to stay within the queue (high absolute values of *p*_10_ in the electronic supplementary material, table S4).

## 5. Movement decisions are less flexible under pressure

We fit our models separately to data obtained in the presence of treatment M to develop an understanding of this treatment's effect on participants' route choices. Figure 2*b* and the electronic supplementary material, table S5, show the results of our model fitting for this data. As in figure 2*a*, we found a global trend of decreasing marginal likelihood from left to right in figure 2*b*. We also found that the same two models as in figure 2*a* had a significantly lower marginal likelihood than the other models (the next closest model had much stronger support, 2log(BF)=10.6). This shows that as before, under treatment M, participants also used information contained in at least one of the model components W, Q and F to choose an exit.

However, in figure 2*b*, it is more difficult to decide reliably which out of these three model components best explained decisions of participants, as the differences in marginal likelihood between models were generally much smaller than in figure 2*a*. Starting from the left-hand side and comparing ‘blocks’ of models as in the previous section shows that there is an overlap between the first and second model blocks and that for the other two model block comparisons, we only find weak positive evidence in support of model blocks to the left (both 2log(BF)<1; e.g. compare model ‘Q, noF, noW, S’ (second block) to model ‘noQ, F, noW, noS’ (third block): 2log(BF)=0.6, from the electronic supplementary material, table S5). It is not the case that a difference in the amount of data used for figure 2*a*,*b* could explain the discrepancy in differences between marginal likelihoods: we used 4746 and 4743 data points for our analysis in the absence and the presence of treatment M, respectively. One possible explanation for the difficulty to distinguish between models could be that under treatment M, participants were less likely to change their mind on which exit to use during the evacuation. Model components Q and F capture changes in the virtual environment over time that appear to inform participants' decision (figure 2*a*). But Q and F also approximate the static difference between exits that is captured in component W. Therefore, if participants respond less to changes in the environment, as suggested by the decrease in *P*(change) under treatment M, it could be more difficult to distinguish between components Q, F and W. Despite this, the global trend in marginal likelihoods in figure 2*b* provides weak positive evidence in support of models that include both components Q and F.

The values of the most likely parameters for our models were similar to the most likely parameter values for the data collected in the absence of treatment M (electronic supplementary material, table S4). Only for the constant component there was a notable difference between the two datasets (parameter *p*_1_). For all models, *p*_1_ took negative values suggesting a slight bias of participants to choose the top exit under treatment M in our experiment (electronic supplementary material, table S5).

## 6. Discussion

We have conducted experiments with over 450 human participants and used a Bayesian model selection approach to explore dynamic route choice mechanisms in interactive simulated crowd evacuations. Our findings suggest that participants tended to base their exit choices on time-dependent information (i.e. differences in queue lengths and queue speeds at exits) rather than on time-independent information (i.e. differences in exit widths or exit route length). When we put participants under additional pressure (treatment M), we still found weak support for similar exit choice mechanisms. But overall participants were less able or willing to adjust their original exit choice in the course of the evacuation under this treatment, confirming earlier findings [14].

Computer-based experiments are no replacement for evacuation drills or observational data from real emergencies. We make no claims that our findings on human route choice in virtual environments also extend to human route choice in real environments. Therefore, our experiment should not be interpreted as a direct test of pedestrian evacuation dynamics in real environments. Instead, our work contributes novel insights into human decision-making via an abstracted route choice task in a virtual environment. Below, we explain in more detail the relevance and limitations of this type of experimental approach, and how the results of such studies are best interpreted.

When considering the information individuals base their route decisions on, it is important to discuss the fact that the top-down view participants had in our virtual environment is likely to differ from how pedestrians visually perceive their environment. The technology for conducting experiments such as ours in more immersive virtual environments already exists (e.g. three-dimensional environment, first person viewpoint [12,21]). However, we argue that the tactical-level decisions we investigate are based on information that humans could detect even if they do not have a top-down view of their environment [14,15]. For example, pedestrians could estimate differences in queue speeds based on heuristics, such as the speed at which a selected person in a queue moves, as suggested previously [9]. In addition, the top-down view and simple controls for interacting with the virtual environment in our experiments served to mitigate differences in computer literacy between participants that can strongly affect results in more immersive virtual environments with more complex controls [21]. In general, virtual environments such as ours facilitate high-throughput behavioural analysis that could be enhanced further by conducting experiments online or on mobile devices [26]. As mentioned in the Introduction, we suggest that considering the expense and potential ethical issues of real evacuation drills with volunteers, experiments such as ours could aid the selection of topics for further study in more life-like experiments from a large set of initial hypotheses.

As indicated earlier, it is possible that the implementation of our experiment could explain the fact that participants did not tend to choose the shorter path when it was made visible (treatment S). It is conceivable that the difference in length between routes was insufficient to produce a clear response in participants or that participants did not realize at the outset of the experiment that the final target of their evacuation was at location T2 (figure 1*a*) and could therefore not determine the shortest exit route. The values of the most likely parameters in our model selection analysis suggest that participants only had a weak propensity to choose the shorter path when it was visible. It is entirely plausible and important in the context of crowd evacuations to hypothesize that the absolute value of this parameter and therefore its influence on individuals' route decisions is likely to depend on the extent to which individuals are familiar with their environment and the shortest exit routes. For example, a more obvious highlighting of the final exit (e.g. using a sign), or even of the shorter route in the virtual environment could lead to a stronger response of participants to this experimental treatment. A detailed investigation of the extent to which this suggestion holds is an important topic for future research.

Introducing an asymmetry into the virtual environment by making one of the exits wider (treatment W) strongly affected participants' exit choices. Our high-level analysis suggested that participants were significantly more likely to choose the wider exit, but it was only through model selection that we could establish that participants followed the dynamic information provided by differences in queue lengths or queue speeds at the exits rather than the static information provided by differences in exit widths or route length.

Putting additional pressure on participants (treatment M) affected individuals' decision-making in line with previous findings [14]. These results therefore provide considerable evidence to suggest that under conditions similar to treatment M, individuals' decision-making becomes less dynamic and individuals are highly likely to stick with their original decision. This is especially significant in the light of the comparatively small emotional effect treatment M (a motivational message, no reward) is likely to have on individuals. In real emergencies, individuals experience considerably higher levels of stress and anxiety [4,6] and we suggest that it is therefore highly important to continue research into the effect of stress on tactical-level decision-making in real crowd evacuations.

One fundamental difference between our experiments and real crowd evacuations lies in the movement dynamics at the start of the evacuation. Our simulated pedestrians react instantaneously at the start of the evacuation and quickly establish queues at both exits, leaving participants to choose between two exits with queues already in place. This could be compared to the situation when a pedestrian enters a room and is faced with a decision between two already used exits. In real crowd evacuations from a single room, the initial dynamics of queue formation are likely to be important. For example, people may join the queue that is growing faster (following others). Our experiment does not allow us to study this phenomenon here, but there is a considerable body of work on decision-making in animal groups that could provide a useful starting point [27–29]. For example, following others [27] and isolation-aversity [28] could be important factors in determining individuals' route choices. In this context, it is also interesting to compare our findings on the relative influence of the length and the movement of queues at exits on individuals' decisions to a recent model selection study which suggests individuals are more likely to respond to recent movements of others rather than responding to the size of aggregations [29]. The model selection we use here could also be used to study queue formation events and more generally it could be applied to different types of data, such as individual decisions during evacuation trials or even in real emergencies. This would also help to address the question of the extent to which our findings can be extrapolated to real crowd evacuations.

While we cannot guarantee that our findings accurately reflect the route choices of pedestrians in real environments, they provide an empirically founded starting point for algorithms modelling dynamic route choice in humans that is different from ad-hoc implementations of individual-level decisions (e.g. [9]). Such algorithms are important for designing simulation models for crowd movement in egress that are already routinely used in building and event planning [6]. However, it is important to note that any simulation model used in this way requires validation against empirical data on real crowd movement [4,6].

One connection that we have not made explicitly here, but that could be of interest for future work, is to express dynamic route choice of pedestrians in the framework of mathematical queuing theory [30,31]. In this framework, bottlenecks, such as exits, would represent servers with queues forming in front of them. The key differences to classical mathematical queueing theory in this scenario would be that the waiting times in queues depend on the level of demand (flow at bottlenecks depends on pedestrian density [32]) and that the arrival time for queues could also depend on the queue at the server relative to the queues at other available servers (e.g. as indicated by our work).

In conclusion, we provide evidence that participants in our computer-based evacuations used dynamic information to make exit route choices. This suggests that in principle participants are likely to react to changing circumstances, rather than only following static information provided by features of the built environment. We also show that this dynamic flexibility in decision-making is reduced when participants are put under additional pressure, providing a potent reminder for the importance of considering how the motivational and emotional state of individuals may affect the dynamics of crowd evacuations.

## Supplementary Material

Supplementary Information: Supplementary methods and results.

## Data accessibility

The data presented in this paper are available from the Dryad Digital Repository (http://doi.org/10.5061/dryad.7m645).
