# Supplementary material for: Information use by humans during dynamic route choice in virtual crowd evacuations
Source: R Soc Open Sci. 2015 Jan 21;2(1):140410. doi: 10.1098/rsos.140410 (PMC4448793; doi:10.1098/rsos.140410)
Supplement: Supplementary Information: Supplementary methods and results. [file rsos140410supp1.pdf]

Supplementary information

Information use by humans during dynamic route choice in virtual crowd evacuations

Nikolai W. F. Bode, Armel U. Kemloh Wagoum, Edward A. Codling

1. Supplementary methods

1.1 Virtual environment

To study what type of information influences the exit route choice of individuals, we followed previously established methodology and asked human participants to take part in interactive computer-simulated crowd evacuations [1, 2]. During the experiment, participants had a top-down view of a virtual environment. Ninety simulated pedestrians were moving inside this environment and participants controlled the movement of one pedestrian using mouse clicks. The environment comprised of a central room that was connected to an exit point by two corridors of unequal length (see figure 1 in main text). The extent to which participants could see the global layout of the environment depended on the experimental treatment (see section 1.3 below), but in general, participants could only see the movement dynamics of other pedestrians inside the room they were occupying. Our custom-built computer program recorded the movement of all simulated pedestrians, as well as the timing and on-screen location of human participant's mouse clicks. At the start of the experiment, participants received instructions on how to steer their pedestrian and were given the option to provide their age and gender (see section 1.2 below for full details). Subsequently, participants had to complete two consecutive tasks in the virtual environment.

The first task served to familiarise participants with the control of their pedestrian and the virtual environment. Participants were positioned at a fixed starting position inside the central room of the environment (see figure 1A in main text). They then had to follow the direction marked by arrows and steer their pedestrian around an obstacle towards a fixed target location (T1 in figure 1A in main text). The obstacle was a straight wall that

separated the central room into two parts, leaving small gaps at the top and bottom of the wall, as seen in the lower half of figure 1C in the main text. We randomly assigned participants to navigate around the top or bottom of this symmetrical obstacle to avoid inducing a directional bias. The obstacle was included for training purposes only and was removed in the second task. During the first task the simulated pedestrians moved randomly inside the central room and the adjoining corridors. To avoid revealing information about the nature of the experimental treatments, the exits connecting the central room to the corridors were initially blocked. All participants successfully completed this training task and we did not use data recorded during this task in our analysis.

As soon as participants had reached their first target, the second task was initiated. A message instructing participants that there had been an accident and that they should leave the virtual building was displayed for five seconds. Subsequently, the exits connecting the central room to the corridors opened and the simulated pedestrians started to perform an evacuation by exiting the central room to reach the exit from the virtual environment (T2 in figure 1A in main text). The simulated crowd split approximately evenly between the two exits from the central room. In this task, participants had to choose between the top and bottom exit to leave the central room and complete the experiment by moving to target T2. Recall that one route to target T2 was always longer than the other route (C1 is longer than C2; figure 1A in main text). To avoid introducing a directional bias in the asymmetry of the environment, we flipped the environment through its horizontal axis so that C1 (the longer route) was accessed via the bottom exit and the shorter route, C2, via the top exit.

Throughout the experiment, participants were allowed to ask questions on how to steer their pedestrian. Ethical approval for our experiment was granted by the Ethics Committee of the University of Essex.

1.2 Full instructions given to participants

In this section we provide the full instructions displayed during the experiment, as well as verbal information given to participants. All instructions were provided in German. To save space, we only report the translated instructions. The standard instructions of how to interact with the virtual environment follow previously established experimental protocol [1, 2].

Before commencing the experiment, participants saw a screen with the following instructions: *“Hello, in this*

game you are a person in a building with many other people. You can steer the person you are playing with the mouse. Simply click on the location you want to move to. To start with, follow the red arrows to your first target – a green circle. The person you are playing is represented by a black filled circle. To begin, press the START button above." After starting the game, all participants could see the following message in a panel underneath the simulated environment: *"Follow the red arrows to the target."*

Participants were also given additional verbal instructions at the start of the experiment: *"You are the black dot here, and when you click somewhere, this is where you are going to move."* and *"Don't worry about the other people, you can bump into them."*

At the start of the simulated evacuation, participants either saw a message stating *"Attention, there has been an accident. Leave the building!"*, or, under treatment M, they saw the message presented in figure 1 in the main text. After the simulated evacuation had started, participants could see the message, *"Leave the room",* in the panel underneath the simulated environment in the absence of treatment M. Under treatment M, participants saw a different message that is described in the main text.

1.3 Experimental treatments

Manipulating the virtual environment allowed us to precisely control the information available to participants and the situation experienced by participants. Each participant was subjected to one out of a total of eight different experimental conditions. The eight different experimental conditions were as follows: control treatment, three primary treatments, three pairwise combinations of primary treatments, combination of all three primary treatments. We describe the control treatment and the three primary treatments in detail below. Participants were allocated a unique number which was incremented by one between consecutive participants. Assigning experimental conditions to participants according to modulo 8 of this number ensured an even split of participants across experimental conditions. To ensure participants did not exchange information on the experimental conditions, they were not allowed to watch others before taking part in the experiment and after taking part, they were not allowed to talk to others who had not yet taken part.

The experimental treatments are illustrated in figure 1 in the main text. In the control treatment, participants could not see the global layout of the virtual environment and the simulated crowd split approximately evenly

between exits. The environment participants experienced was therefore symmetrical (figure 1B; see below on details of how simulated pedestrians split between exits).

In contrast to the control treatment, participants could see the global layout of the environment in the 'shortest path' treatment (S; figure 1A in main text). This treatment was designed to test whether participants plan ahead and realise that the two corridors are of unequal length, making the exit route via C2 shorter than the route via C1.

The 'motivation' treatment (M) was similar to an experimental treatment previously used to assess the effect putting additional pressure on participants had on their route choice [1]. Instead of only instructing participants to exit the central room at the start of the second task, participants were encouraged to do so as quickly as possible and to beat a current fastest evacuation time (figure 1C in main text). In addition, a counter displaying participant's current time and the fastest time were displayed throughout the second task. For consistency of this treatment, the fastest time displayed on screen was not updated, even if participants evacuated faster.

Finally, the 'exit width' treatment (W) introduced a different type of asymmetry to the virtual environment in addition to the difference in length between the two corridors. In this treatment, the exit leading to the longer corridor, C1, was one-and-a-half times as wide as the opposite exit (figure 1D in main text). When the environment was flipped through its horizontal axis (see above), the location of the wider exit under treatment W moved with the location of the longer route C1. With this treatment, we were particularly interested to investigate whether participants responded to the static asymmetry in the environment or to resulting differences in the movement dynamics of the simulated pedestrians in front of the exits. For example, the length of the pedestrian queue in front of the wider exit is likely to decrease faster.

1.4 Simulated crowd evacuation

We simulated pedestrian movement in the virtual environment in the same way as in previous work on this subject [1, 2]. While pedestrians moved in continuous two-dimensional space, their interactions with the environment (e.g. walls) and movement preferences (e.g. towards target) were encoded in a discrete floor field, as previously proposed [3]. Interactions between pedestrians (e.g. collision avoidance, body

compression forces) and interactions between pedestrians and the environment (e.g. avoiding walls, movement towards target) were modelled as forces acting on point masses, following previous theoretical work [4]. Pedestrian movement was simulated by numerically solving the resulting equations of motion for each simulated pedestrian, incrementing time in fixed simulation update steps that corresponded to about 5ms of real time. We chose fixed parameter values for our simulations that ensured participants had enough time to respond to the dynamics. The full details of our simulation model can be found in [1, 2].

Initially, the simulated pedestrians were distributed evenly over the central room and the two corridors. The simulation was paused when the message was displayed at the start of the second task (5 seconds). During the second task, all pedestrians in the top half of the central room exited through the top exit and all pedestrians in the bottom half through the bottom exit. As pedestrian movement in the first task was random, this led to an approximately even distribution of pedestrians across exits. Once pedestrians reached the final target, they were removed from the simulation and the graphic display.

1.5 Data collection and preliminary analysis

Our experiment was conducted alongside a large-scale study on pedestrian dynamics in Düsseldorf, Germany (19th – 22nd June 2013; project details: www.basigo.de). We recruited participants from the paid volunteers who were already taking part in separate experiments. A total of 480 individuals participated in our experiment, each of them only once. Our results are based on the data from 464 participants. The remaining 16 participants had accidentally terminated the computer program before all data was saved to files. A total of 388 (84%) participants reported their age (median: 23 years; mean: 24.38 years; youngest: 18 years; oldest: 64 years) and 432 (93%) participants reported their gender (224 male, 208 female, 32 unwilling to disclose). We did not record data on nationality or ethnicity. The split of participants across treatments was as follows: control (58); S (58); M (57); W (56); M and S (58); W and S (59); W and M (58); W, M and S (60).

For a preliminary, high-level analysis, we recorded two binary summary statistics for each participant: *P(shortest path)*, whether or not participants used the shorter path in the virtual environment, and *P(change)*, whether or not participants changed their choice of exit during the simulated evacuation. For the latter measure, *P(change)*, we recorded a change of mind for participants if they walked for at least one fifth of the height of the central room towards one exit before changing direction and exiting through the opposite exit.

We used generalised linear models (GLMs) to assess the effect of our experimental treatments on these summary statistics (also see figure S1 for participants' responses). These statistical tests and all following analysis were conducted in the R programming environment, version 3.0.2 [5]. The GLMs we fit to our data had binomial error structure and included one of the two summary statistics as the response variable. The explanatory variables were the three primary experimental treatments (W, S, and M; categorical), gender (categorical) and age (continuous). Both GLMs included an intercept. We set a significance threshold of $p<0.05$, and report all p-values from single parameter tests (parameter value equals zero under the null hypothesis). Due to missing data for age and gender, 78 data points could not be included in this analysis.

1.6 Model selection

The model selection approach was inspired by previously established methodology [6]. We modelled *P(top exit|y(t))*, the probability of individuals to choose the top exit conditioned on the function *y(t)* that captured time-dependent and time-independent aspects of the virtual environment (recall that the top exit can lead to the shorter route via C2 or to the longer route via C1, as in figure 1A in main text). For simplicity of presentation we hereafter drop the time dependence in our notation for *y*. The general structure of our models is given by the following equation:

$$P(\text{top exit}|y)=1 / (1 + \exp(y)), \qquad \text{(S1)}$$

where the details and parameters of the model are contained in the function y. We chose this model structure to ensure that P(top exit|y) took values between zero and one and that $P(\text{bottom exit}|y) = 1 - P(\text{top exit}|y) = 1/(1+\exp(-y))$. For simplicity, we specify our models in terms of *P(top exit|y)* and refer the reader to the simple change in sign that is necessary to obtain *P(bottom exit|y)*.

The function *y* differed across models and determined which aspects of the virtual environment were included in the model. For simplicity, we assumed that *y* is a linear combination of the contributions of different components, where each component captures the effect of one aspect of the virtual environment. In the simplest case, $y = p_1$, where p_1 is a constant and a model parameter. In practice, y included a number of different components relating to the difference in door widths, the difference in exit route lengths, the difference in the lengths of simulated pedestrian queues in front of exits, and more. The different components

included in our models and their implementations are detailed in table S1.

We used data at fixed intervals of 50 simulation update steps in our analysis (about 0.25s; e.g. 1000 simulation update steps led to 20 data points for our analysis). Let $V_{i,t}$ equal one if participant i chose the top exit at time t and equal to zero if participant i chose the bottom exit. We determined $V_{i,t}$ by using the on-screen location of participants' mouse clicks in the virtual environment. Mouse clicks in the top half of the screen indicated a decision to walk towards the top exit and vice-versa. To avoid recording ambiguous decisions, we excluded mouse clicks that were within a vertical distance of one fifth of the central room height from the horizontal line through the centre of the screen (which coincided with the vertical centre of the central room). If there was no change in decision or no additional mouse clicks, consecutive values of $V_{i,t}$ had the same value. For each participant, we used all data from the first included mouse click after the start of the second task until the participant pedestrian had left the central room. In principle, it is possible that this procedure could lead to misclassifications of participants' decisions. For example, consider the situation when a participant is located at the side of a queue and wants to move to the back of the queue. If the participant clicked into the opposite half of the room to move to the back of the queue, this would be recorded as a change in decision. We checked the data visually, to ensure such scenarios did not occur.

Let ***D*** be our data set. Under the assumption that instantaneous decisions only depend on the current state of the virtual environment (see also [6]), the likelihood for a given parameter set θ of model X is given by:

$$P(\mathbf{D}|\ \theta,X) = \Pi_i\ \Pi_t\ [P(top\ exit|y)V_{i,t} + P(bottom\ exit|y)(1-\ V_{i,t})], \qquad \text{(S2)}$$

where the products Π_i and Π_t run over all participants i and all data output steps for each participant, respectively. The function y depends on t, θ and i. The marginal likelihood of X, $P(\mathbf{D}|X)$, is found by integrating over the space of unknown parameters, Θ:

$$P(\mathbf{D}|X) = \int_\Theta P(D|\ \theta,X)P(\theta|X)d\ \theta, \qquad \text{(S3)}$$

where $P(\theta|X)$ is the assumed prior distribution of the parameters for X. As we have no prior knowledge for parameter values, we choose uniform prior distributions and use the same distributions across all models to

allow for a fair comparison between models [6]. The prior distributions for our model parameters are given in table S1.

The numerical integration of equation S3 was performed via annealed importance sampling [7]. Briefly, we started with 500 samples from the prior distribution and moved via 20 intermediate distributions from the prior distribution to the target distribution, $P(\mathbf{D}|\theta,X)$. For each intermediate distribution we performed 200 Markov chain transitions (Metropolis-Hastings acceptance criterion). The intermediate distributions were defined by a relative weighting, ω, of the target distribution to the prior distribution, where: $\omega=(k/20)^4$, $k=0,1,\ldots,20$. We omit the precise details of this procedure and refer the reader to the literature on this topic [7, 8]. The numerical integration was repeated five times for each model, to obtain an indication of the uncertainty in our estimate for the marginal likelihood. We additionally report the most probable parameter values for each model, averaged across the 5 replicate integrations [6].

2. Supplementary figure

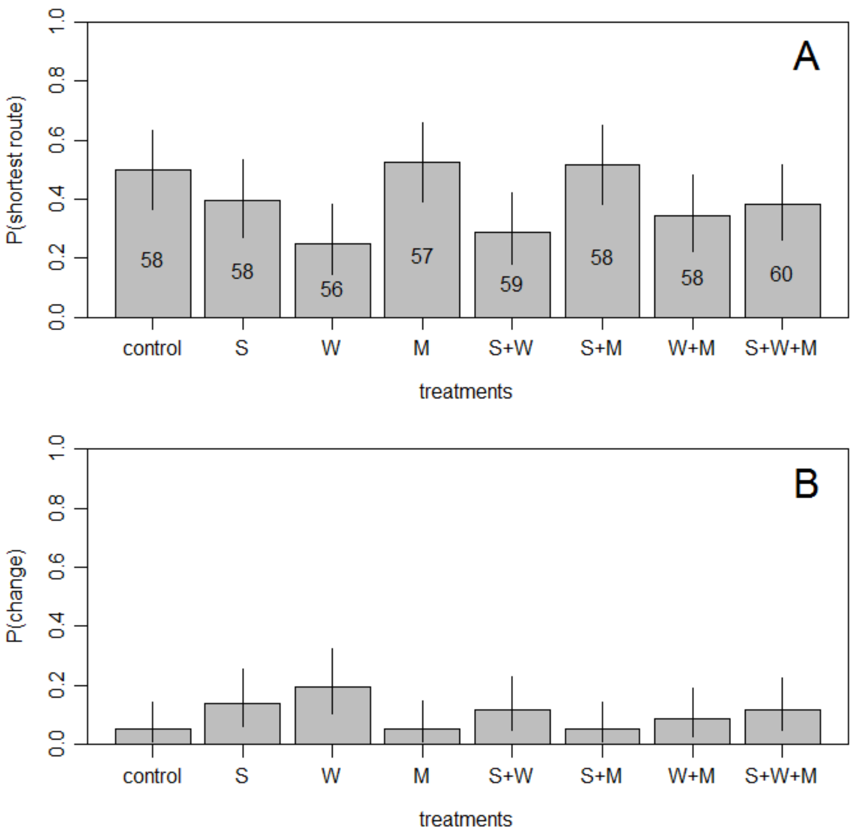

Figure S1: proportion of participants who chose the shortest route in the simulated evacuations, *P(shortest route)*, panel A, and the proportion of participants who changed their mind on which exit to use during the evacuation, *P(change)*, panel B, for the different experimental treatments. When two or more primary treatments are present, this is denoted by a '+'. Numbers inside the bars in A indicate the number of participants per treatment. Error bars show 95% confidence intervals for the observed probabilities. The GLMs were fitted to all data (see section 1.5 above). For example, in panel B, even if *P(change)* does not change noticeably from the control condition to treatment M, overall, across all experimental conditions, treatment M has a statistically significant effect (see table S3 below).

3. Supplementary tables

Table S1: Summary of model components and parameters. Implementation details are given with respect to equation S1 in the SI text. The function *y* in a particular model is given by the sum of the components that are included in the model. Model parameters are denoted by p_i, $i=1,\ldots 10$. The last column gives the prior distribution for the model parameters that was used in the Bayesian model comparison ($U_c(0,10)$ denotes a continuous and $U_d(0,10)$ a discrete uniform distribution over the interval $[0,10]$). To give an example, for a model without the components for Q or F, but with all other components, we have $y = p_1 + p_2\ \alpha + p_3\ \beta + p_8\ \varepsilon(p_9) + p_{10}\ \zeta_{top}$ (short description of this model: noQ, noF, W, S; cf figure 2 in main text). The sign of model parameters indicates the effect of a model component. For example, $p_2>0$ implies that in equation 1, $P(top\ exit|y)$ increases if the top route is shorter ($\alpha=-1$) and vice-versa.

Short name	Contribution to *y*	Description	Prior distribution
Constant	p_1	Baseline constant.	$p_1 \sim U_c(-1,1)$
S, shortest route	$p_2\ \alpha$	Under treatment S, α encodes which route is shorter: $\alpha=-1$ top route, $\alpha=1$ bottom route. Under treatment noS, $\alpha=0$.	$p_2 \sim U_c(-1,1)$
W, door widths	$p_3\ \beta$	Under treatment W, β encodes which exit is wider: $\beta=1$ top exit, $\beta=-1$ bottom exit. Otherwise, $\beta=0$.	$p_3 \sim U_c(-1,1)$
Q, queue lengths	$p_4 <\gamma_{top}(t) - \gamma_{bottom}(t)>_{p5}$	Difference between the number of simulated pedestrians at the top (γ_{top}) and at the bottom exit (γ_{bottom}). The brackets $<\ldots>_{p5}$ denote the average over the p_5 preceding data output steps.	$p_4 \sim U_c(-1,1)$, $p_5 \sim U_d(2,10)$
F, queue speeds	$p_6\ [\delta_{top}(p_7) - \delta_{bottom}(p_7)]$	Difference in rate of change of queue lengths, calculated over p_7 data output steps: $\delta_{top}(p_7) = \gamma_{top}(t-p_7) - \gamma_{top}(t)$.	$p_6 \sim U_c(-1,1)$, $p_7 \sim U_d(2,10)$
Distances from exits	$p_8\ \varepsilon(p_9)$	ε is the distance of the participant pedestrian from the top exit minus its distance from the bottom exit. All differences in distances smaller or equal to p_9 are set to zero.	$p_8 \sim U_c(-1,1)$, $p_9 \sim U_c(0,10)$
Jammed in queue	$p_{10}\ \zeta$	ζ is the number of pedestrians blocking participant's route to the opposite exit (positive when participant has chosen the top exit and vice-versa). ζ is computed as the number of simulated pedestrians within a two-pedestrians-wide band pointing from the current participant position to the opposite exit. The band is truncated at the centre of the central room.	$p_{10} \sim U_c(-10,10)$

Table S2: Testing the effect of the primary treatments on *P(shortest route)*. Binomial GLM (logit link function); response variable: Boolean indicating whether participants use shorter route to exit the central room. The table shows the estimate, standard error and statistical test results for the explanatory variables in the model. Significant values are shown in bold.

**Effect**	**Estimate**	**s.e.**	**z value**	**P**
**intercept**	**-0.99**	**0.47**	**-2.08**	**0.04**
S	-0.04	0.21	-0.21	0.83
**W**	**-0.78**	**0.21**	**-3.63**	**0.0003**
M	0.34	0.21	1.57	0.12
gender	-0.04	0.21	-0.18	0.86
**age**	**0.03**	**0.02**	**2.04**	**0.04**

Table S3: Testing the effect of the primary treatments on *P(change)*. Binomial GLM (logit link function); response variable: Boolean indicating whether participants changed their mind on which exit to use. The table shows the estimate, standard error and statistical test results for the explanatory variables in the model. Significant values are shown in bold.

**Effect**	**Estimate**	**s.e.**	**z value**	**P**
intercept	-1.65	0.89	-1.86	0.06
S	0.13	0.34	0.39	0.69
W	0.57	0.35	1.63	0.10
**M**	**-0.71**	**0.36**	**-1.97**	**0.048**
gender	0.44	0.35	1.27	0.21
age	-0.04	0.03	-1.01	0.31

Table S4: Model comparison and parameter estimates for data without the M treatment. We show averages over 5 replicate numerical integrations of equation S3 in the SI text. Models are described in terms of the components that are included in the model (e.g. model 'Q,noF,W,S' includes components Q, W and S, but not component F). Parameters p_5 and p_7 indicate the time-scale over which participants observed differences in the dynamics or sizes of the queues in front of the exits.

**Model**	**Log P(D|M)**	**Const.**	**S**	**W**	**Q**		**F**		**Distances from exit**		**In jam**
		p_1	p_2	p_3	p_4	p_5	p_6	p_7	p_8	p_9	p_{10}
Q,F,W,S	-427.3	0.09	0.37	0.01	0.09	8.4	-0.16	10	0.99	0.04	-6.72
Q,F,noW,S	-426.3	0.08	0.37	-	0.09	8.6	-0.16	10	0.99	0.05	-7.25
Q,F,W,noS	-429.2	0.12	-	0.17	0.09	8.2	-0.16	10	0.99	0.04	-6.74
Q,F,noW,noS	-428.3	0.08	-	-	0.09	8.4	-0.14	10	0.99	0.04	-7.80
Q,noF, W,S	-432.8	0.03	0.35	-0.42	0.10	2.0	-	-	0.99	0.05	-6.94
Q,noF,noW,S	-434.2	0.10	0.24	-	0.12	2.0	-	-	0.98	0.06	-7.81
Q,noF,W,noS	-433.9	0.06	-	-0.27	0.10	2.0	-	-	0.99	0.07	-6.49
Q,noF,noW,noS	-433.9	0.11	-	-	0.12	2.0	-	-	0.98	0.05	-7.68
noQ,F, W,S	-438.2	-0.2	0.38	-0.48	-	-	-0.14	10	0.99	0.05	-5.71
noQ,F,noW,S	-439.5	-0.16	0.27	-	-	-	-0.18	10	0.99	0.07	-6.90
noQ,F,W,noS	-440.2	-0.19	-	-0.30	-	-	-0.14	10	0.99	0.10	-6.35
noQ,F,noW,noS	-439.9	-0.15	-	-	-	-	-0.17	10	0.99	0.07	-8.04
noQ,noF, W,S	-444.5	-0.3	0.37	-0.96	-	-	-	-	0.99	0.11	-7.82
noQ,noF,noW,S	-463.4	-0.26	-0.03	-	-	-	-	-	0.92	0.05	-8.31
noQ,noF,W,noS	-445.9	-0.28	-	-0.79	-	-	-	-	0.96	0.11	-7.33
noQ,noF,noW,noS	-461.7	-0.27	-	-	-	-	-	-	0.92	0.06	-8.69

Table S5: Model comparison and parameter estimates for data under the M treatment. We show averages over 5 replicate numerical integrations of equation S3 in the SI text. Models are described in terms of the components that are included in the model (e.g. model 'Q,noF,W,S' includes components Q, W and S, but not component F). Parameters p_5 and p_7 indicate the time-scale over which participants observed differences in the dynamics or sizes of the queues in front of the exits.

**Model**	**Log P(D|M)**	**Const.**	**S**	**W**	**Q**		**F**		**Distances from exit**		**In jam**
		p_1	p_2	p_3	p_4	p_5	p_6	p_7	p_8	p_9	p_{10}
Q,F,W,S	-325.4	-0.55	0.29	-0.15	0.06	7.2	-0.09	10	0.99	0.06	-7.22
Q,F,noW,S	-325.0	-0.50	0.30	-	0.06	6.8	-0.11	10	0.99	0.06	-6.42
Q,F,W,noS	-326.2	-0.54	-	-0.01	0.05	7.6	-0.09	10	0.99	0.07	-6.94
Q,F,noW,noS	-325.4	-0.53	-	-	0.05	7.0	-0.09	10	0.99	0.07	-7.99
Q,noF, W,S	-325.4	-0.60	0.34	-0.43	0.06	2.8	-	-	0.99	0.06	-7.61
Q,noF,noW,S	-326.6	-0.54	0.15	-	0.08	2.0	-	-	0.99	0.07	-7.68
Q,noF,W,noS	-325.8	-0.58	-	-0.24	0.06	2.2	-	-	0.99	0.07	-6.80
Q,noF,noW,noS	-325.8	-0.52	-	-	0.07	2.0	-	-	0.99	0.07	-6.63
noQ,F, W,S	-327.8	-0.66	0.24	-0.24	-	-	-0.10	10	0.99	0.07	-6.70
noQ,F,noW,S	-327.5	-0.62	0.17	-	-	-	-0.13	10	0.99	0.08	-7.99
noQ,F,W,noS	-327.4	-0.65	-	-0.16	-	-	-0.10	10	0.99	0.08	-6.30
noQ,F,noW,noS	-326.9	-0.63	-	-	-	-	-0.11	10	0.99	0.08	-7.75
noQ,noF, W,S	-328.2	-0.75	0.26	-0.66	-	-	-	-	0.99	0.09	-5.88
noQ,noF,noW,S	-334.8	-0.69	-0.10	-	-	-	-	-	0.99	0.08	-7.07
noQ,noF,W,noS	-328.1	-0.73	-	-0.51	-	-	-	-	0.99	0.08	-8.16
noQ,noF,noW,noS	-333.5	-0.70	-	-	-	-	-	-	0.99	0.08	-7.73

References

1. Bode NWF, Codling EA. 2013 Human exit route choice in virtual crowd evacuations. *Anim. Behav.* **86**, 347-358.

2. Bode NWF, Kemloh Wagoum AU, Codling EA. 2014 Human responses to multiple sources of directional information in virtual crowd evacuations. *J. R. Soc. Interface* **11**, 20130904.

3. Burstedde C, Klauck K, Schadschneider A, Zittartz J. 2001 Simulation of pedestrian dynamics using a two-dimensional cellular automaton. *Physica A* **295**, 507-525.

4. Helbing D, Farkas I, Vicsek T. 2000 Simulating dynamical features of escape panic. *Nature* **407**, 487-490.

5. R Core Team. 2012. R: A language and environment for statistical computing. *R Foundation for Statistical Computing, Vienna, Austria*. ISBN 3-900051-07-0, URL http://www.R-project.org/.

6. Mann RP et al. 2013 Multi-scale Inference of Interaction Rules in Animal Groups Using Bayesian Model Selection. *PLoS Comput Biol.* **9**, e1002961.

7. Neal RM. 2001 Annealed importance sampling. *Statistics and Computing* **11**, 125-139.

8. Friel N, Pettitt AN (2008) Marginal likelihood estimation via power posteriors. *J. R. Stat. Soc. B.* **70**, 589-607.
